# Rebleeding After Aneurysmal Subarachnoid Hemorrhage in Two Centers Using Different Blood Pressure Management Strategies

**DOI:** 10.3389/fneur.2022.836268

**Published:** 2022-02-21

**Authors:** Lionel Calviere, Celine S. Gathier, Marie Rafiq, Inez Koopman, Vanessa Rousseau, Nicolas Raposo, Jean François Albucher, Alain Viguier, Thomas Geeraerts, Christophe Cognard, Gabriel J. E. Rinkel, Mervyn D. I. Vergouwen, Jean-Marc Olivot

**Affiliations:** ^1^Stroke Unit, CHU Toulouse, Toulouse, France; ^2^Toulouse Neuroimaging Center, INSERM, UPS, Toulouse, France; ^3^Department of Neurology and Neurosurgery, UMC Utrecht Brain Center, University Medical Center Utrecht, Utrecht University, Utrecht, Netherlands; ^4^MeDatAS-CIC, CIC1436, Centre Hospitalier Universitaire, Toulouse, France; ^5^Department of Anesthesiology and Critical Care, CHU Toulouse, Toulouse, France; ^6^Department of Neuroradiology, CHU Toulouse, Toulouse, France

**Keywords:** rebleeding, intracranial aneurysm, subarachnoid hemorrhage, blood pressure, delayed cerebral ischemia

## Abstract

**Background:**

High systolic blood pressure (SBP) after aneurysmal subarachnoid hemorrhage (aSAH) has been associated with an increased risk of rebleeding. It remains unclear if an SBP lowering strategy before aneurysm treatment decreases this risk without increasing the risk of a delayed cerebral ischemia (DCI). Therefore, we compared the rates of in-hospital rebleeding and DCI among patients with aSAH admitted in two tertiary care centers with different SBP management strategies.

**Methods:**

Retrospective cohort study. Consecutive patients from Utrecht and Toulouse admitted within 24 h after the aSAH onset were enrolled. In Toulouse, the target SBP before aneurysm treatment was ≤140 mm Hg, while, in Utrecht, an increased SBP was only treated in extreme situations. We compared SBP levels, the incidence of rebleeding within 24 h after admission, and DCI during hospitalization.

**Results:**

We enrolled 373 patients in Utrecht and 149 in Toulouse. The mean SBP on admission was similar but lower in Toulouse 4 h after admission (127.3 ± 17.4 vs. 138. ± 25.7 mmHg; *p* < 0.0001). After a median delay of 3.7 h (IQR, 2.3–7.4) from admission, 4 patients (3%) in Toulouse *vs*. 29 (8%) in Utrecht experienced a rebleeding. After adjustment for Prognosis on Admission of Aneurysmal Subarachnoid Hemorrhage (PAASH) score, aneurysm size, age, and delay from ictus to admission, the HR was 0.66 (95% CI: 0.23–1.92). Incidence of DCI was 18% in Toulouse and 25% in Utrecht (adjusted OR, 0.68; 95% CI: 0.41–1.11).

**Conclusion:**

Our results suggest that an intensive SBP lowering strategy between admission and aneurysm treatment does not decrease the risk of rebleeding and does not increase the risk of DCI compared to a more conservative strategy.

## Introduction

In patients with aneurysmal subarachnoid hemorrhage (aSAH), rebleeding is the most feared early complication (1–4). It occurs in 8 to 23% of patients within the first 72 h after ictus, and is associated with a 60% case-fatality rate (4–6). Currently, emergent aneurysm treatment remains the only effective strategy to prevent rebleeding, but often it is not possible due to logistical reasons. Moreover, it has been shown that most of the acute rebleeding occurs within the first hours after the onset. Thus, additional strategies aiming to reduce this risk might be of particular interest during the acute management of aSAH before aneurysm repair.

Several factors have been associated with a high rebleeding risk: high systolic blood pressure (SBP), neurological deficit, and/or a decreased level of consciousness on hospital admission, aneurysm size, and location (5–9). Among these factors, high SBP is the only modifiable one. High SBP could increase aneurysmal transmural pressure and, therefore, increase the risk of rebleeding (10). But the relationship between an SBP threshold and recurrent bleeding risk is not established (7, 8, 10, 11). No randomized controlled trial (RCT) has assessed the impact of blood pressure, lowering on the risk of rebleeding, and a prolonged SBP lowering has been suspected to favor the occurrence of a delayed cerebral ischemia (DCI) (12). As a consequence, there is uncertainty whether lowering SBP during the first hours after aSAH could reduce the risk of rebleeding and is well-tolerated (13, 14). It explains that there is no consensus in the literature as evidenced by the different recommendations of European Stroke Organization and American Heart and Stroke Association (13, 14).

Therefore, we aimed to compare the rates of rebleeding and DCI in a comparative retrospective historical cohort study of patients with aSAH enrolled in two tertiary care centers using different SBP management strategies between admission and aneurysm repair.

## Patients and Methods

We conducted a retrospective cohort study of consecutive patients with aSAH from two tertiary comprehensive stroke centers: Utrecht (the Netherlands) and Toulouse (France). The patients were enrolled between October 2013 and October 2015 in Toulouse, and January 2009 and December 2014 in Utrecht. Inclusion criteria were: (1) adult patients with aSAH; and (2) admission in the two tertiary comprehensive stroke centers within 24 h after the onset. The patients with non-aneurysmal SAH were excluded.

### Blood Pressure Management Strategies

In Toulouse, since 2013, neuro-intensivists have applied a strict SBP management protocol from admission to aneurysm treatment, targeting an SBP of ≤140 mmHg. After aneurysm treatment, SBP thresholds are relaxed as recommended to reduce the risk of DCI. Only extreme SBP values >200 mmHg are decreased to maintain the SBP between 140 and 200, with an objective of 100-110 of mean arterial BP. The first line medication to reduce the SBP is oral (12 tablets of 30 mg/day) or continuous IV nimodipine (2.5 mg/h). If necessary, IV urapidil (5 mg by cc) is added to achieve the SBP goals, starting with a bolus of 25 mg of urapidil, then an increase of 0.5 cc/h by 0.5 cc/h every 15 min until the blood pressure objective is achieved (max 6 cc/h = 30 mg/h). Nicardipine is added if the goal is not achieved (1 mg/h then increase of 0.5 mg/h every 15 min with a maximum of 6 cc/h = 3 mg/h). In Utrecht, elevated blood pressures before aneurysm treatment are usually not treated, unless values are extreme (usually SBP above 180–200 mmHg), which is decided on an individual basis. Oral, but not IV, nimodipine is prescribed to every patient with aSAH according to current recommendations (13).

Concerning the other complications of aSAH, the patients were similarly managed in both centers according to the ESO guidelines (13).

### Data Collection

The following data were collected: age, sex, pre-admission oral anticoagulant therapy, history of hypertension (defined by high blood pressure medication at admission), Glasgow Coma Scale (GCS) score on admission, Prognosis on Admission of Aneurysmal Subarachnoid Hemorrhage (PAASH) score on admission (15, 16), size and location of the ruptured aneurysm measured on digital-subtracted angiography or CT angiography, delay between ictus and hospital admission, and delay between hospital admission and beginning of aneurysm treatment. Ictus was defined as the acute headache or the neurological deterioration related to aneurysm rupture. It was reported by the patient or the family.

Data on systolic BP were collected on admission and every 2 h until aneurysm treatment, death, or rebleeding. The BP was recorded by a radial line or by cuff measurements, depending on ICU or stroke unit admission.

### Endpoints

The primary endpoint was the occurrence of in-hospital rebleeding within 24 h after admission. It was defined by a sudden clinical deterioration with signs of a new or an increased hemorrhage on CT scan compared with previous CT imaging, or found at autopsy, or a sudden clinical deterioration suspect for rebleeding with fresh blood in the ventricular drain among those for whom no CT scan or autopsy was obtained.

Secondary endpoint was clinical deterioration due to a delayed cerebral ischemia (DCI) according to consensual definition: occurrence of focal neurological impairment (such as hemiparesis, aphasia, apraxia, hemianopia, or neglect), or a decrease of at least 2 points on the Glasgow Coma Scale (either on the total score or on one of its individual components). This deficit had to last for at least 1 h, was not immediately apparent after aneurysm occlusion, and could not be attributed to other causes by means of clinical assessment; Transcranial Doppler and CT angiogram were used to confirm a proximal vasospasm. The CT or MRI scanning of the brain confirmed the presence of acute ischemic lesion (17). Monitoring for DCI was performed during the ICU stay of the patients (from 2 weeks to 1 month).

According to the French ethic and regulatory law (public health code) on retrospective studies based on the exploitation of usual care, the data do not have to be submitted to an ethics committee, but they have to be declared or covered by reference methodology of the French National Commission for Informatics and Liberties (CNIL). According to the General Data Protection Regulation, this study completed all the criteria. It was approved by Toulouse University Hospital board and was registered in the register of retrospective studies of the Toulouse University Hospital (register No.: RnIPH 2020-10) and covered by the MR-004 (CNIL No.: 2206723 v 0). The ethics board of the UMC Utrecht decided that no formal assessment was needed because, for this analysis of already collected data, there was no need to approach the patients or collect additional data.

### Statistics

In descriptive analyses, means, and standard deviations (SD) or medians and interquartile ranges (IQR) were presented for quantitative variables. For qualitative variables, numbers and percentages were used.

We first compared the baseline characteristics of patients with aSAH between the two centers. Qualitative variables were compared using Chi^2^ Test (or Fisher's exact test as appropriate) and quantitative variables using Wilcoxon–Mann–Whitney test.

To compare the SBP according to time between centers, a mixed model for repeated measures was performed. This model was chosen to overcome the missing data at different measurement times. This model accounted for several SBP measurements over time (non-independent data) and a time-center interaction. Sensitivity analysis was performed during the period when 75% of the rebleedings occurred (during the first 8 h after admission).

Kaplan–Meier curves were generated for a time to an in-hospital rebleeding within 24 h after admission. The two curves representing the two centers were compared using Log-Rank test. The starting point was the time of ictus. The event of interest was the in-hospital rebleeding within 24 h after admission, and the patients were censored if the aneurysm treatment or death occurred within 24 h after admission. If more than one episode of rebleeding occurred, we used the time of the first rebleeding. Cox proportional hazard regression was used to calculate the hazard ratios (HR) with 95% confidence intervals (CI). The HRs were adjusted for differences in baseline characteristics between the two centers and established risk factors of rebleeding.

To estimate the association between the center and the occurrence of clinical deterioration due to DCI, a logistic regression model was used. First, a crude odds ratio (OR) was calculated using the center without a protocol for lowering the active blood pressure (Utrecht) as the reference group. Then, the OR was adjusted for age, sex, size of aneurysm, and PAASH score on admission.

All tests were two sided and considered statistically significant at α level of 0.05. All statistical analyses were conducted using an SAS® Software, version 9.4.

## Results

### Baseline Characteristics

A total of 522 patients were included (149 in Toulouse and 373 in Utrecht). In Utrecht, the patients were older, had a higher PAASH score, a larger aneurysm, and a shorter delay from ictus to admission. The median delay from admission to aneurysm treatment was longer in Utrecht [19 h (IQR: 12–27) vs. 15 h (IQR: 6–21), *p* < 0.0001] (Table 1).

**Table 1:** Characteristics of the patients from Utrecht and Toulouse.

	**Centers**	
	**Toulouse *N* = 149**	**Utrecht *N* = 373**
Age [mean, (+/–SD), years]	54 (14.8)	57.6 (12.8)
Women (%)	93 (62.4)	266 (71.3)
PAASH score on admission (median, IQR)	1 (1–2)	2 (1–4)
PAASH score (%)		
1	85 (57.1)	116 (31.1)
2	28 (18.8)	123 (32.9)
3	6 (4.0)	27 (7.2)
4	21 (14.1)	54 (14.5)
5	9 (6.0)	53 (14.2)
Aneurysm size [mean, (+/–SD), mm]	6.8 (3.7)	7.7 (4.3)
Hypertension (%)	54 (36.2)	122 (32.7)
Delay between ictus and admission [mean, (+/–SD), hours]	7.6 (6.1)	5.4 (6.0)
Delay between admission and aneurysm treatment (median, IQR, hours)	15 (6–21)	19 (12–27)
Aneurysm treatment within 24 h after admission (%)	120 (80.5)	215 (57.6)
SBP on admission [mean (+/–SD), mmHg]	148.4 (26.6)	148.5 (30.0)

*IQR, interquartile range; SBP, systolic blood pressure*.

*Statistics for qualitative variables: n (%) for quantitative variables: mean (SD) or if notified: median (IQR)*.

### Evolution of Systolic Blood Pressures

**Figure 1:**
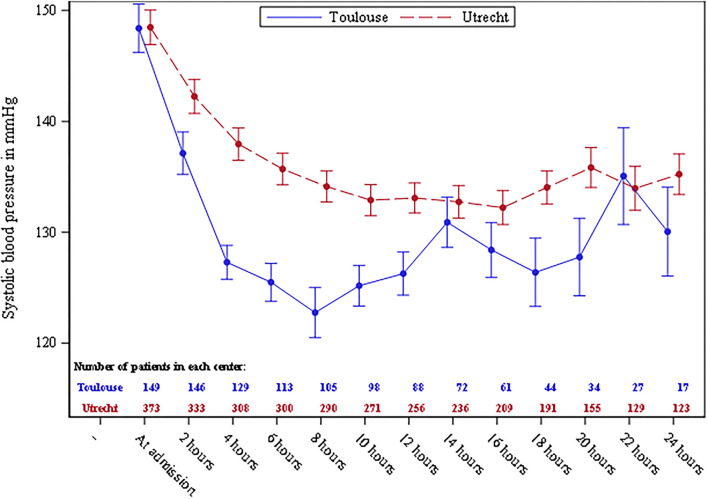
Systolic blood pressure (mmHg) distribution among the two centers during the first 24 h after admission. Plot means with standard error bars. The patients are censored after aneurysm treatment or death.

SBP was similar in both centers at admission. The difference of mean SBP between the 2 centers reached its maximum 4 h after admission: 127.3 ± 17.4 vs. 138 ± 25.7 mmHg; *p* < 0.0001 (Table 2, Figure 1). The mixed model for repeated measures showed that SBP decreased over time in the whole study population [beta = −1.3 (SD = 0.27), *p* < 0.0001]. This decrease was more pronounced in Toulouse compared to Utrecht [beta = −5.9 (SD ± 2.1), *p* = 0.007]. No interaction between the center and time was observed [beta = 0.29 (SD ± 0.30), *p* = 0.33].

**Table 2:** Evolution of systolic blood pressure.

	**Toulouse**	**Utrecht**	
	***N* = 149**	***N* = 373**	
	**Mean (SD)**	**Mean (SD)**	*p* value^*^
SBP at admission (mmHg)	148.4 (26.6)	148.5 (30.0)	0.89
SBP 4 h after admission (mmHg)	127.3 (17.4)	138.0 (25.7)	<0.0001
Last SBP measured (before aneurysm treatment, death, rebleeding or at 24 h) (mmHg)	128.6 (20.8)	137.4 (24.9)	0.0003
Crude delta SBP between admission and after four hours (mmHg)	−21.8 (29.1)	−10.4 (28.5)	0.0003

*SBP, systolic blood pressure*.

* *Wilcoxon Mann–Withney Test*.

### Rebleeding

**Figure 2:**
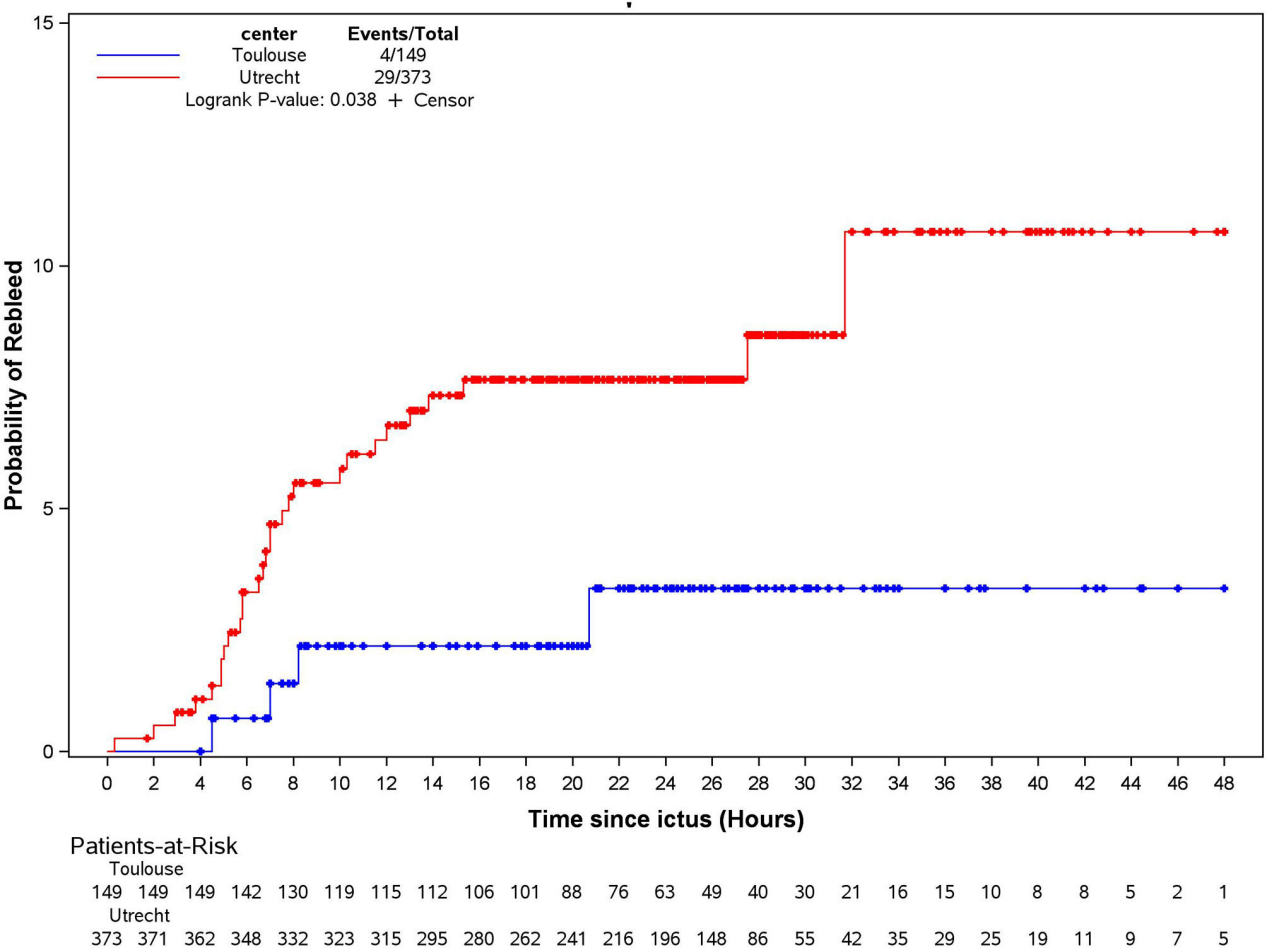
Kaplan–Meier one minus survival curves of time to rebleeding occurrence.

Thirty-three patients (6.3%) experienced rebleeding within 24 h after admission, after a median delay of 3.7 h (IQR, 2.3–7.4). Rebleeding occurred in 29 (8%) patients in Utrecht and in 4 (3%) in Toulouse [crude HR: 0.35 (95% CI: 0.12–0.99)]. After adjustment for PAASH score, aneurysm size, age, and delay from ictus to admission, the hazard ratio was 0.66 (95% CI: 0.23–1.92). The Kaplan–Meier curve is shown in Figure 2.

As 75% of the acute recurrent bleedings occurred within 8 h after admission, sensitivity analysis was performed during this period. The SBP decreased significantly over time [beta = −6.0 (SD = 0.64), *p* < 0.0001], although no difference was observed between the two centers [beta = −2.2 (SD = 2.5), *p* = 0.37]. However, an interaction between the center and time was observed [beta = −2.72 (SD = 0.75), *p* = 0.0003], which means that SBP decreased faster in Toulouse than in Utrecht.

### Delayed Cerebral Ischemia

Clinical deterioration due to DCI occurred during the hospitalization in 25% of the patients in Utrecht vs. 18% in Toulouse, crude OR: 0.65 (95% CI: 0.40–1.03). After adjustment for age, sex, PAASH score, and aneurysm size, the OR was 0.68 (95% CI: 0.41–1.11).

## Discussion

This is, to our knowledge, the first study comparing occurrence of in-hospital rebleeding and DCI in comprehensive stroke centers, applying two different emergent SBP management strategies. Our results show that the intensive BP lowering strategy applied in Toulouse resulted in a faster BP reduction and lower SBP values than in Utrecht during the first hours after admission. However, after adjustment for confounders, the occurrence of acute rebleeding before aneurysm repair was similar in both centers. An important finding of our study was that an intensive BP lowering strategy was not associated with an increased risk of DCI.

Our results confirm that in hospitals, acute recurrent bleeding of within 24 h after admission of patients with aSAH is low, in centers which were able to propose an expedited aneurysm treatment like ours. However, most instances of in-hospital rebleeding occurred within the first 8 h after admission, a period during which the difference in SBP level was the highest between the two centers. But these rates remain low, and, if the absolute rate of rebleeding was almost reduced to 50% between the two centers, the HR was similar after adjustment for confounders because of unbalanced baseline characteristics between the two centers. Moreover, if the rate of in-hospital acute rebleeding was substantial (3–6%), it may remain too low to demonstrate the impact of a potentially effective strategy that could, in addition to emergent aneurysm repair, have an impact on a patient outcome. Therefore, our results do not support the hypothesis that intensive SBP treatment decreases the risk of rebleeding.

One previous retrospective, monocentric cohort study performed after the implementation of an intensive SBP reduction strategy (SBP target 140/90 mmHg) reported a low rate (3.1%) of recurrent bleeding, but there was no comparative cohort (18). Another monocentric retrospective study compared two cohorts of 104 patients with an aSAH during two time periods: one period with a BP management protocol (SBP target 160 mmHg) and one without this protocol (19). It showed a non-significant decrease in the incidence of in-hospital rebleeding after implementation of the SBP control period. Another study showed that BP management targeting SBP <160 mmHg and DBP <100 mmHg was associated with a lower incidence of rebleeding but a higher incidence of DCI (12). However, that study was performed 30 years ago, during which, delays between ictus and aneurysm treatment were longer. In addition, the patients received an antihypertensive treatment for the duration of their hospitalization, exposing them to an increased risk of DCI favored by decreased BP. In our study, the patients in Toulouse underwent BP reduction within 48 h after aneurysmal rupture, followed by a liberal management after aneurysm treatment according to current international recommendations. We hypothesize that the timing and short duration of intensive SBP management might explain the absence of an increased risk of DCI in our study. Regarding the occurrence of DCI, there are, to our knowledge, no studies that specifically investigated the relationship between pre-aneurysmal treatment short duration (<48 h after aneurysm rupture) of an aggressive SBP management and DCI.

Interestingly, not only the intensive SBP lowering protocol in Toulouse resulted in a significant reduction of SBP but also the more conservative management protocol in Utrecht. We speculate that the application of European recommendations by themselves (including oral nimodipine, pain management, urinary catheter, etc.) contributed to this reduction that remained slower and less pronounced than in Toulouse.

We need to address a few limitations. First, unexpectedly, risk factors of rebleeding differed between the two centers. In Utrecht, aneurysm size was larger and PAASH score higher, and the patients were admitted earlier after ictus and treated later than in Toulouse, exposing them to a longer period at high risk of in-hospital rebleeding. As a consequence, these imbalances induced a major bias, limiting the assessment of the potential efficacy of the intensive SBP-lowering strategy. In addition, some critical factors in the risk of rebleeding and/or DCI, such as the amount of blood in the subarachnoid space on initial imaging, were not recorded.

Second, the study participants were taken from historical cohorts that were collected a few years ago. This limitation came from our center in France for several reasons: first, this database was collected for a short period of time during which we implemented this SBP strategy and evaluated its safety. In addition, since then, our group has participated in RCT, using drugs that are susceptible to direct interfere with DCI and rebleeding. Therefore, our data collection was strictly limited to this time period. However, to our knowledge, early aSAH management recommendations have not been modified since then, and, therefore, we consider that our findings apply to our current practice.

Third, we studied the effect of an intensive BP-lowering strategy initiated in a tertiary referral center. Since the risk of rebleeding is highest in the first few hours after ictus, many instances of rebleeding may have occurred during transfer between the primary hospital where the diagnosis of SAH was made, and the tertiary referral center. It may be that initiation of an intensive BP-lowering strategy may result in a decreased risk of rebleeding if initiated in the primary hospital prior to hospital transfer. Future prospective studies aiming to assess these strategies should probably expand their enrollment to the pre-hospital phase in transfer patients and/or to centers where the resources to propose emergent aneurysm treatment are limited.

In conclusion, our results suggest that an intensive SBP-lowering strategy between admission and aneurysm treatment does not decrease the occurrence of acute rebleeding but also does not increase the risk of DCI. Future studies are needed to investigate if this strategy is applied during a longer period, and, in a more balanced population, it is associated with an improved functional outcome.

## Data Availability Statement

The raw data supporting the conclusions of this article will be made available by the authors, without undue reservation.

## Ethics Statement

According to the French Ethic and Regulatory Law (public health code) retrospective studies based on the exploitation of usual care, data do not have to be submitted to an Ethic Committee but they have to be declared or covered by reference methodology of the French National Commission for Informatics and Liberties (CNIL). According to the General Data Protection Regulation, this study completed all the criteria. It was approved by Toulouse University Hospital Board and was registered in the register of retrospective studies of the Toulouse University Hospital (number's register: RnIPH 2020-10) and cover by the MR-004 (CNIL number: 2206723 v 0). The ethics board of the UMC Utrecht decided that no formal assessment was needed because for this analysis of already collected data there was no need to approach patients or collect additional data.

## Author Contributions

LC, MR, CG, GR, J-MO, and MV: conception, contributed to interpretation of data, and drafted the manuscript. VR contributed to the statistical plan and statistical analysis. MR, CG, IK, LC, JA, AV, NR, CC, and TG collected data, contributed to interpretation of the results, and made critical revisions to the manuscript. The authors approved the final version of the manuscript.

## Conflict of Interest

Unrelated to this study, JA received a travel grant, speaker fees, or research funding grant from Bayer, Bristol Myers Squibb. LC received a travel grant, speaker fees, or research funding grant from Boehringer Ingelheim, Pfizer. NR received a travel grant, speaker fees, or research funding grant from Fullbright Fundation, Harvard University and Philippe Foundation. CC received a travel grant, speaker fees, or research funding grant from Medtronic Cerenovus StrykerMIVI Neuroscience, Sensome, Microvention. J-MO received travel grant, speaker fees, or research funding grant from Abbvie, Aptoll, Medtronic, Bristol Myers Squibb. The remaining authors declare that the research was conducted in the absence of any commercial or financial relationships that could be construed as a potential conflict of interest.

## Publisher's Note

All claims expressed in this article are solely those of the authors and do not necessarily represent those of their affiliated organizations, or those of the publisher, the editors and the reviewers. Any product that may be evaluated in this article, or claim that may be made by its manufacturer, is not guaranteed or endorsed by the publisher.
